# Multi-Level Socioenvironmental Contributors to Childhood Asthma in New York City: a Cluster Analysis

**DOI:** 10.1007/s11524-021-00582-7

**Published:** 2021-11-29

**Authors:** Sana Khan, Sarah Bajwa, Diksha Brahmbhatt, Stephanie Lovinsky-Desir, Perry E. Sheffield, Jeanette A. Stingone, Sheng Li

**Affiliations:** 1 City University of New York Institute for State and Local Governance, New York, NY USA; 2 NYC Department of Health and Mental Hygiene, New York, NY USA; 3 Columbia University Irving Medical Center, New York, NY USA; 4 Icahn SOM at Mt Sinai, New York, NY USA; 5 Columbia University, New York, NY USA; 6 City University of New York School of Public Health, New York, NY USA

**Keywords:** Health equity, Environmental health, Asthma, Cluster analysis

## Abstract

Childhood asthma exacerbation remains the leading cause of pediatric emergency department visits and hospitalizations and disproportionately affects Latinx and Black children, compared to non-Latinx White children in NYC. Environmental exposures and socioeconomic factors may jointly contribute to childhood asthma exacerbations; however, they are often studied separately. To better investigate the multiple contributors to disparities in childhood asthma, we compiled data on various individual and neighborhood level socioeconomic and environmental factors, including education, race/ethnicity, income disparities, gentrification, housing characteristics, built environment, and structural racism, from the NYC Department of Health’s KIDS 2017 survey and the US Census’ American Community Survey. We applied cluster analysis and logistic regression to first identify the predominant patterns of social and environmental factors experienced by children in NYC and then estimate whether children experiencing specific patterns are more likely to experience asthma exacerbations. We found that housing and built environment characteristics, such as density and age of buildings, were the predominant features to differentiate the socio-environmental patterns observed in New York City. Children living in neighborhoods with greater proportions of rental housing, high-density buildings, and older buildings were more likely to experience asthma exacerbations than other children. These findings add to the literature about childhood asthma in urban environments, and can assist efforts to target actionable policies and practices that promote health equity related to childhood asthma.

## Introduction

In New York City (NYC), childhood asthma remains the leading cause of pediatric emergency department visits and hospitalizations [1]. Asthma is a clear example of childhood health disparities, as it disproportionately affects Latinx and Black children, compared to non-Latinx White children [2, 3]. Socioenvironmental and housing factors, both on the individual and community levels, increase the likelihood of childhood asthma and asthma exacerbation and are the main drivers of health disparities [4–14]. However, environmental conditions and socioeconomic factors are not commonly studied together in studies of childhood asthma exacerbation. This is a fundamental gap in research as Latinx and Black children in urban environments are more likely to experience social and environmental asthma triggers simultaneously, potentially increasing the burden of disease [15, 16]. Identifying the patterns of socioenvironmental factors that affect children living in urban environments is a first step toward determining if these patterns are associated with greater risk of asthma exacerbations. Such research can lead to more holistic policies and interventions that simultaneously target multiple factors to address asthma disparities in urban environments.

Statistical regression models are the primary analytic approach used in the majority of asthma studies. Given high correlations between the socioenvironmental factors present in the urban environment, it is difficult to use traditional regression models to simultaneously investigate patterns of exposure. In contrast, unsupervised machine learning approaches do not consider the outcome of interest nor do they rely on the investigator’s hypothesized relationships between features. Instead, unsupervised machine learning uses data-adaptive methods to uncover patterns between features within high-dimensional exposure data. Unsupervised approaches, such as clustering, group individuals with similar exposure patterns can be interpreted to identify the features that most strongly characterize the specific patterns. Once individuals are grouped into pattern-based clusters, it is possible to compare the incidence of health outcomes of interest across clusters. Clustering enables the identification of the collection of exposures that contribute to the greatest risk of outcomes of interest and can inform—the development of multifactorial interventions and policies [17–20].

The objective of this study was to analyze the roles of socioenvironmental and housing factors at both the individual and neighborhood levels in NYC that, in combination, contribute to childhood asthma exacerbations. To better understand individual and community level exposures associated with asthma exacerbation, we used a two-step approach of unsupervised clustering and logistic regression to identify exposure patterns and examine associations between clusters and asthma exacerbations.

These results will help inform strategic, community-level interventions by understanding specific co-occurring factors that families and neighborhoods are exposed to instead of focusing on factors individually known to be associated with asthma. This research supports the utilization of an equity lens by focusing on structural changes in housing and environments needed to address disparities in health, safety, and asthma exacerbation. By identifying such patterns and associations, policies and programs that help manage and reduce health disparities related to childhood asthma can be strengthened and leveraged across disciplines.

## Methods

### Data Collection and Study Population

Data used in this study came from the NYC KIDS survey conducted by the NYC Department of Health and Mental Hygiene in 2017 [21]. The NYC KIDS survey collected data via telephone interviews with 7507 randomly selected households with one or more children 0–13 years of age. Respondents were parents, guardians, or other family members who were sufficiently knowledgeable about a selected child’s health, doctor visits, and general activities, as well as family and neighborhood characteristics. Most respondents (84.0%) were the mothers of the children. The overall response rate was 19.6%, and the overall cooperation rate for completion of the survey was 57.4%. The current analysis was restricted to children 6 years and older (*n* = 2157) due to potential inaccuracies in clinical diagnosis of asthma in children under 6 years. Thirty-two (1.5%) of 2157 respondents with a missing or invalid zip code were excluded due to an inability to identify neighborhood-level values. Of the 2125 remaining study participants, 7.8% had at least one missing value for the individual-level exposure variables. The respondents with and without missing values did not show significant differences on socioeconomic and housing characteristics. Therefore, 92.2% of respondents with complete data for all variables were used in the analyses (*n* = 1959).

### Exposure Variables

Individual-level socioenvironmental and housing factors were included from the 2017 NYC KIDS Survey (see Table 1). Neighborhood-level variables were created using 5-year zip code-level estimates from the 2013–2017 American Community Survey (ACS). These factors reflected demographics, education level, income disparities, markers of gentrification, building characteristics, and an indicator of structural racism. Income disparities were categorized by the Gini coefficient [22]. Gentrifying neighborhoods in NYC were identified by zip code as previously published [23]. The definition of structural racism used in this analysis was constructed, as previously published, by identifying the racial inequity (ratio of Black to white population) in college degree attainment and unemployment rate [24]. Neighborhood-level variables constructed from the ACS estimates were appended to 2017 NYC KIDS respondents by zip code. Individual-level factors indicate exposure in the 12 months prior to the survey and neighborhood-level factors represent average exposures over the last 3 years.

**Table 1:** Descriptive characteristics of the sample, 6–13 years, 2017 NYC KIDS

	Full population	Lifetime diagnosis of asthma		Among children with asthma*	
	N=1959	Asthma: 324	No asthma: 1635	Asthma attack in the past 12 months (*N* = 128)	No asthma attack in past 12 (*N* = 194)
Age of child (mean, SD, years)	9.2(2.4)	9.3(2.3)	9.2(2.4)	9.1(2.2)	9.4(2.3)
Sex of child (%)					
Male	52	57.4	50.9	62.5	54.1
Female	48.0	42.6	49.1	37.5	45.9
Race/ethnicity of child (%)					
White, non-Latinx	21.7	6.5	24.7	6.3	6.7
Black, non-Latinx	21.2	26.2	20.2	30.5	23.2
Latinx	41.2	57.4	38	50.8	61.9
Asian/PI, non-Latinx	11.3	6.2	12.3	7	5.7
Other, non-Latinx	4.5	3.7	4.7	5.5	2.6
Borough					
The Bronx	21.4	31.5	19.4	33.6	29.9
Brooklyn	33.4	23.8	35.4	26.6	22.2
Manhattan	13.4	12	13.6	13.3	10.8
Queens	24.7	25.3	24.5	19.5	29.4
Staten Island	7.1	7.4	7	7	7.7
Age of parent 1 (%)					
16 to 24	1.2	2.2	1	1.6	2.6
25 to 44	72.3	72.8	72.2	66.4	76.8
45 to 64	25.3	22.8	25.8	28.1	19.6
65 or older	1.2	2.2	1	3.9	1
Highest household education (%)					
Less than high school	10.8	14.5	10.1	14.1	14.9
High school degree or GED	22.6	26.9	21.7	22.7	29.4
Some college	20.9	22.5	20.6	24.2	21.6
College graduate	45.7	36.1	47.6	39.1	34
Household composition					
One parent, no other adults	18.9	26.5	17.4	29.7	24.7
Both parents (w/ or w/o other adults)	59.9	44.4	62.9	39.8	47.9
At least one parent with 2 + adults	17.5	22.8	16.4	23.4	21.6
Does not live with parent	3.7	6.2	3.2	7	5.7
Household poverty %FPL (%)					
< 100%	37.4	48.5	35.2	42.2	52
100–199%	23.9	24.7	23.7	24.2	25.3
200–399%	15.5	13.4	15.8	14.1	13.4
400–599%	11.1	7.7	11.7	11.7	5.2
600 + %	12.1	5.6	13.5	7.8	4.1
Public assistance (%)					
Yes	34.7	48.8	31.9	48.4	48.5
No	65.3	51.2	68.1	51.6	51.5

^*^Two children were missing data on asthma exacerbation question so sample size does not match total number of children who reported an asthma diagnosis

### Outcome Variables

Asthma exacerbation was determined by using the 2017 NYC KIDS survey question: “During the past 12 months, has your child had an episode of asthma or an asthma attack?” Lifetime asthma was determined with the question “Has a doctor or health professional ever told you or another caregiver that your child has asthma?” Asthma exacerbation, instead of lifetime asthma, prevalence, was the primary outcome of this analysis. The lifetime asthma measure did not indicate whether asthma was current or historical and was less accurate in assessing temporal causal associations with recent housing and environmental conditions. Numerous studies use parental report to classify asthma exacerbation. The consistency and reliability of parental report have been validated [25].

### Statistical Analysis

The demographic, socioeconomic, and household characteristics of all respondents for children aged six to 13 years from 2017 NYC KIDS were first examined in univariate analysis for the total study population.

Cluster analysis was then used to identify exposure patterns in socioenvironmental level characteristics. Modifiable housing and neighborhood factors were included, and unmodifiable individual-level factors, such as age, race/ethnicity, and sex, were excluded from the cluster analysis. Asthma outcomes were also excluded because the goal was to identify patterns across the housing and neighborhood-level characteristics matrix (a social/built environment phenotype) and not predict asthma status. Collinearity between features was controlled by removing less modifiable factors within highly correlated pairs. This resulted in removing two features, “ < 1 occupant per room at home” and “percent of household with kids in a zip code area.”

Due to the presence of both continuous and categorical variables, k-prototype clustering from Clustmixtype package in *R* [26] was applied. The k-prototypes algorithm has the ability to handle mixed data types by combining the k-means algorithm for continuous data and the k-modes algorithm for categorical data. It is an iterative algorithm, similar to k-means. It begins by initializing individuals to random cluster prototypes, calculates the distance from each observation to the clusters, assigns observations to the closest clusters, and then updates the cluster prototypes for all features. This algorithm repeats until clusters have stabilized (i.e., individuals do not change cluster assignment after recalculation of distance) or the maximum number of iterations has been reached. The distance metric that dictates how observations are assigned to clusters combines Euclidian distance for the continuous features with simple matching distance for the categorical features. The weight of continuous vs categorical features is controlled by the tuning parameter, lambda. Lambda was calculated directly from the data. The number of clusters, k, is set by the investigator. We selected k by first using the data-driven Elbow method to determine the number of clusters that balances minimizing the variance within clusters by selecting a parsimonious number of clusters [27]. Once the data-driven optimal k was selected, clustering was repeated and compared when varying the number of clusters by 2 in either direction (i.e., k-2, k-1, k_1, k + 2). We then examined the composition of the individual features across these values of k to determine the k-value that produced the clusters that were most interpretable and notably different in terms of the mean values of the socioenvironmental features. Cluster membership using the final version of k was recorded for each individual in the study.

Next, the association between cluster membership and asthma exacerbations was assessed using multiple logistic regression. Confounders were individual-level variables that could influence both the features of a child’s neighborhood or housing environment, as well as their risk of asthma exacerbations. These variables include the following: the child’s race/ethnicity, sex, age, household composition, and household poverty.

All the statistical analyses were implemented in R (version 3.5.2) [28].

## Results

### Study Participant Characteristics

In the 2017 NYC KIDS survey, 1959 respondents had children between the ages of six and 13 years and complete data. The average age of these children was 9.2 years (SD = 2.4), with an approximately even distribution of males and females. The racial/ethnic distribution was 41.2% Latinx, 21.2% non-Latinx Black, 21.7% non-Latinx White, 11.3% non-Latinx Asian/Pacific Islander, and 4.5% other (Table 1).

Of these children, 16.5% had a parental report of diagnosis of asthma by a health professional at any given point in their life. Among children with a diagnosis of asthma, more than half were Latinx (57.4%), a majority came from low-income households at < 200% of the federal poverty level (73.6%), and 39.5% experienced an asthma exacerbation in the past 12 months (Table 1). Most children with asthma exacerbations were Latinx (50.8%) or non-Latinx Black (30.5%) and were male (62.5%). Table 1 displays complete participant characteristics by asthma diagnosis and recent asthma exacerbation.

### Cluster Identification

**Fig. 1:**
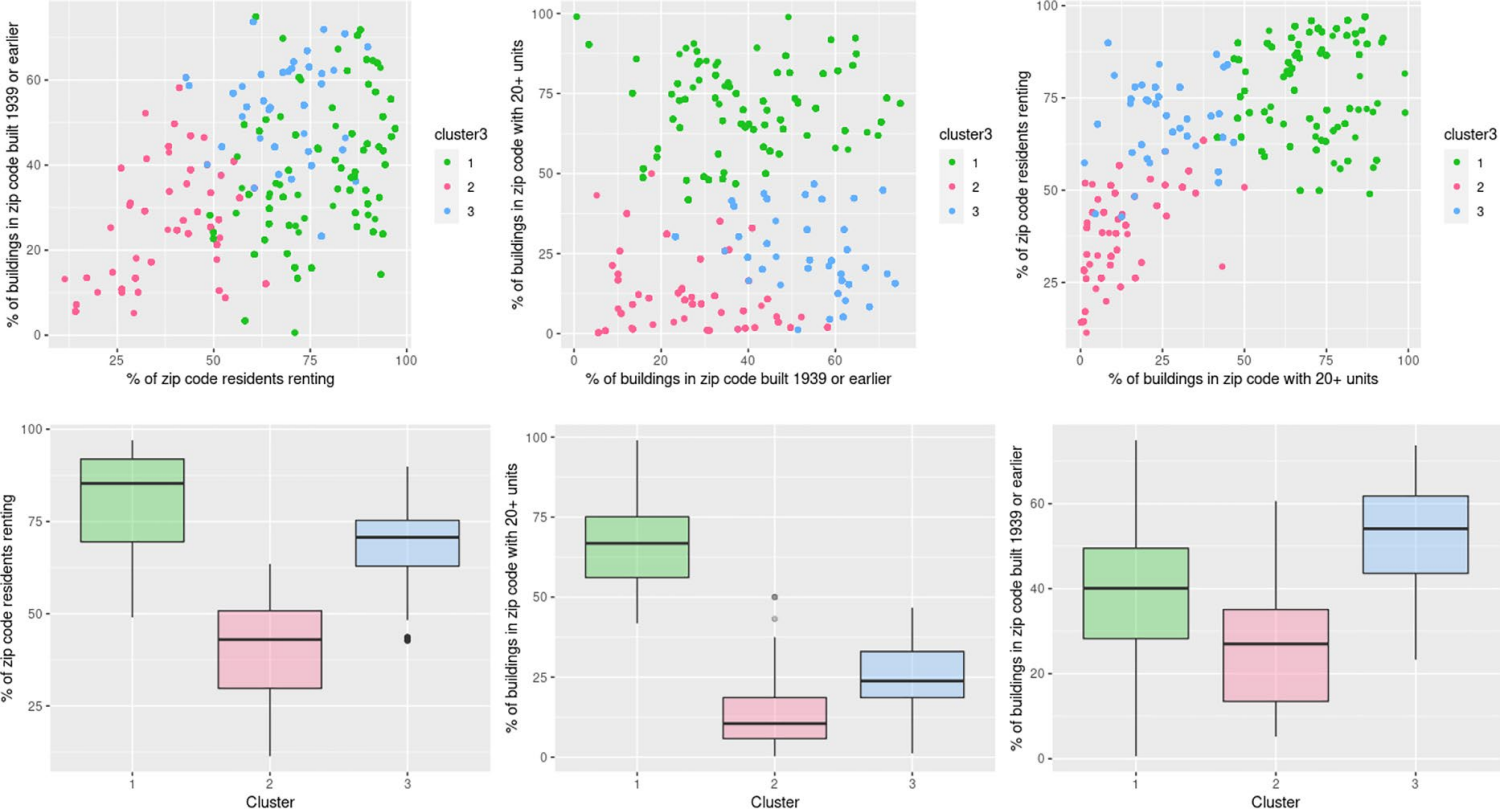
Cluster distribution by three most important features driving clustering. **a**–**c** Distribution of children by cluster according to the three most important features driving clustering. **d**–**f** Mean proportions of these three housing characteristics within each cluster. *Three most important cluster driving features: (1) percent of renters within a zip code area; (2) percent of buildings built before 1940 in the zip code; and (3) percent of buildings with 20 or more units in the zip code. *Three identified clusters: (1) high-density renters cluster; (2) newer, less dense buildings cluster; (3) older, less dense buildings cluster

The cluster analysis indicated that three clusters produced interpretable patterns in housing and neighborhood-level characteristics. The primary features driving clustering were all at the neighborhood level. The top three features were as follows: percent of renters within a zip code, percent of buildings built before 1940 in the zip code, and percent of dense buildings (20 or more units) in the zip code. The first cluster consisted of children living in zip codes with a high proportion of renters and dense buildings (*N* = 963), therefore named “high-density renters cluster.” The second cluster had children in zip codes with a low proportion of renters, less dense buildings, and fewer buildings built before 1940 (*N* = 395), labeled “newer, less dense buildings cluster.” The third cluster was notable for buildings that were less dense and older (*N* = 601), labeled “older, less dense buildings cluster.” Fig. 1a–c shows the distribution of children by cluster according to the three most important features. Figure 1d–f shows the mean proportions of these three housing characteristics within each cluster. Children in the high-density renters cluster lived in zip codes where 80.6% of residents rented their homes. This proportion was 40.4% for the newer, less dense buildings cluster, and 69.2% for the older, less dense buildings cluster. The average proportion of dense buildings (20 + units) was 67.0% for the high-density renters cluster, 13.4% for the newer, less dense buildings cluster, and 26.6% for older, less dense buildings cluster. The mean percentage of older buildings (built before 1940) was 52.3% for the older, less dense buildings cluster, 39.9% for the high-density renters cluster, and 26.6% for the newer, less dense buildings cluster.

### Additional Cluster Characteristics

Although the three most important features driving cluster formation were at the neighborhood level, several individual-level demographic and housing characteristics varied according to cluster (Table 2). Almost half (48.2%) of the high-density renters cluster included Latinx children. The newer, less dense buildings cluster consisted of the highest proportion of non-Latinx Black children (26.8%). A quarter (25.3%) of the older, less dense buildings cluster was non-Latinx white. The newer, less dense buildings cluster and the older, less dense buildings cluster included similar proportions of non-Latinx Asian/Pacific Islanders (14.2% and 14.5%), with the lowest percentage in the high-density renters cluster (8.1%).

**Table 2:** Distribution of select characteristics by cluster

	High-density renters cluster (*N* = 963)	Newer, less dense buildings cluster (*N* = 395)	Older, less dense buildings cluster (*N* = 601)
Race/ethnicity of child (%)			
White, non-Latinx	19.1	22.5	25.3***
Black, non-Latinx	20.5	26.8	18.8
Latinx	48.2	30.6	37.1
Asian/PI, non-Latinx	8.1	14.2	14.5
Other, non-Latinx	4.2	5.8	4.3
Borough			
The Bronx	38.5	7.9	3.0***
Brooklyn	15.0	9.4	78.9
Manhattan	27.2	0.0	0.0
Queens	19.3	39.8	17.5
Staten Island	0.0	34.2	0.7
Roach sighting in past 90 days	41.1	17.2	35.1***
Mice sighting in past 90 days	27.3	14.7	26.8***

*X*-square test for overall association between cluster membership and other variables, *, **, and ***, indicate *P*-value < 0.05, < 0.01, and < 0.001

The borough of residence of participants in each cluster varied greatly. Those residing in Manhattan had lower representation in the newer, less dense buildings and older, less dense buildings clusters, while almost no Staten Island residents appeared in the high-density renters or older, less dense buildings clusters. The high-density renters cluster was composed of primarily children in the Bronx (38.5%) and Manhattan (27.2%). The majority of the newer, less dense buildings cluster respondents lived in Queens (39.8%) or Staten Island (34.2%). The older, less dense buildings cluster was mainly comprised of participants from Brooklyn (78.9%).

The highest proportion of respondents reporting a cockroach sighting in the past 90 days was in the high-density renters cluster (41.1%), followed by 35.1% of the older, less dense buildings cluster, and the lowest in the newer, less dense buildings cluster (17.5%). Mice sightings in the past 90 days followed the same order—the high-density renters cluster had the highest at 27.3%, followed by the older, less dense buildings cluster at 26.8% and the newer, less dense buildings cluster at 14.7%.

Although we included income inequality, gentrification, and structural racism variables in the cluster analysis, none of these factors were important features in driving cluster formation.

### Distribution of Asthma Exacerbation among Identified Clusters

Asthma exacerbations within the last 12 months varied in these three clusters. Among children who were ever diagnosed with asthma by a health professional, 73 (43.7%), 33 (40.2%), and 22 (30.1%) experienced an asthma exacerbation in the past 12 months in the high-density renters cluster, in the older, less dense buildings cluster and in the newer, less dense buildings cluster, respectively.

### Association between Asthma Exacerbation and Cluster Membership

As shown in Table 3, we developed logistic regression models to explore the association between asthma exacerbation and cluster membership. The newer, less dense buildings cluster was used as the reference group because it had the lowest prevalence of asthma exacerbation. In the unadjusted model, children in the high-density renters cluster had 1.77 (1.00, 3.18) greater odds of having an asthma exacerbation in the past 12 months than children in the newer, less dense buildings cluster. Children in the older, less dense buildings cluster had 1.47 (0.75, 2.91) greater odds of asthma exacerbation as children in the newer, less dense buildings cluster. When adjusted for the child’s sex, age, and race/ethnicity, the odds of exacerbation increased to 2.21 (1.21, 4.16) for the high-density renters cluster and to 1.75 (0.87, 3.55) for older, less dense buildings cluster compared to newer, less dense building cluster. When models were further adjusted for household poverty and household composition, the odds of exacerbation increased further: 2.33 (1.25, 4.44) for high-density renters cluster and 1.88 (0.88, 3.74) for the older, less dense buildings cluster compared to newer, less dense buildings cluster.

**Table 3:** Logistic regression modeling on asthma exacerbation

Cluster	Unadjusted model OR (95% CI)	Adjusted model OR (95% CI)^a^	Adjusted model OR (95% CI)^b^
High-density renters Cluster	1.77 (1.00, 3.18)	2.21 (1.21, 4.16)	2.33 (1.25, 4.44)
Newer, less dense Buildings cluster	Reference	Reference	Reference
Older, less dense Buildings cluster	1.47 (0.75, 2.91)	1.75 (0.87, 3.55)	1.88 (0.88, 3.74)

^a^Adjusted for child’s sex, age, and race/ethnicity

^b^Adjusted for child’s sex, age, race/ethnicity, household poverty, and household composition

## Discussion

In this cross-sectional study, we used an unsupervised data machine learning analysis approach to examine the relationship between socioenvironmental, and housing factors and recent asthma exacerbations by combining individual survey responses with zip-code level demographic, socioeconomic, and housing characteristics.

### Neighborhood and Individual-Level Factors and Child Asthma Exacerbation by Clusters

Three clusters were formed to assess how both individual-level and neighborhood-level socioenvironmental and housing factors were associated with asthma exacerbation. The three primary features driving the formation of these clusters were all at the zip code level: the proportion of residents renting their homes, the proportion of dense buildings (20 or more units), and the proportion of older buildings (pre-1940). The “high-density renters” cluster—characterized by children living in zip codes with a high proportion of renters and dense buildings—had the highest rate of asthma exacerbation among children with a lifetime diagnosis of asthma, as well as the most roach and mice sightings. The “newer, less dense buildings” cluster, on the other hand, with the lowest proportion of renters, the lowest proportion of dense buildings, and the lowest proportion of older buildings, had the lowest rate of asthma exacerbation and the least roach and mice sightings. Compared to the high-density renters cluster, the “older, less dense buildings” cluster had similarly high proportions of renters but fewer dense buildings and more old buildings. The rates of asthma exacerbation and proportion of roach and mice sightings were similar in the older, less dense buildings cluster and the high-density renters cluster and differed from the newer, less dense buildings cluster. This suggests that neighborhoods with older housing, of varying density, may have greater prevalence of indoor environmental triggers, such as rodents in the home and subsequently greater risk of asthma exacerbations among children living in those buildings.

Our findings complement a previous study [29] that demonstrated exposure and asthma risk differ by urban housing type in NYC, with residents in public housing having a higher prevalence of asthma and higher odds for asthma persistence than residents of private family homes. While we were unable to specifically assess whether the dense buildings reported in the high-density renters cluster included public housing complexes, dense buildings are a well-known feature of public housing in NYC and may represent an actionable area for interventions for asthma exacerbations in those specific communities. Additionally, we found clear patterns of clustering and associated asthma exacerbation risk by borough of residence, reinforcing the utility of a tailored place-based approach targeting structural determinants of health used by the NYC Department of Health and Mental Hygiene [30, 31].

Contrary to a growing body of evidence regarding structural determinants of health [32], our measures of income inequality, gentrification, and structural racism did not emerge as primary drivers of clusters. The metrics used for income inequality and structural racism were assessed within ZIP code inequality, but structural forces may be more likely to cause inequalities across ZIP codes by concentrating deprivation within specific communities. This distinction could explain why these features did not contribute to clustering. For example, there is a high level of residential racial and ethnic segregation in NYC due to housing policies that have played an outsized role in this present-day segregation. Though the 2017 NYC KIDS Survey did not specify which participants lived in homes from the 1940s to 1970s, it is crucial to acknowledge historic “redlining” policies (instituted before the Fair Housing Act of 1968) that contributed to segregation in lower-income neighborhoods with older homes. These policies stemmed from the 1930s, when the Home Owners’ Loan Corporation (HOLC) preferentially refinanced mortgages outside of zones outlined in red based off racial demographics [33]. Past studies show that those who have lived in a redlined zone have less access to resources, including health care services [34], and experience higher outdoor air temperatures (micro-urban heat islands) due to the type of building materials common in these areas [33]. Both reduced health care service access and exposure to urban heat islands are associated with poorer asthma outcomes [35–37]. These examples underscore the mechanistic links between policies influencing the built environment and the complexity of disentangling race and ethnicity from these structural determinants.

### Limitations in Socioenvironmental and Housing Exposures and Asthma Outcomes

Our findings are limited by the inability to assess several socioenvironmental and housing exposures that have well-established associations with childhood asthma but were not assessed in the 2017 NYC KIDS Survey. This includes poor ventilation, air pollution, mold, dampness, and exposure to lead. Another housing characteristic we were unable to assess was whether families resided in public or private housing. The surveys provided zip codes of households rather than geocoded addresses of residential spaces, therefore, giving an imprecise housing characterization of the participants’ homes. Additionally, neighborhood characteristics such as proximity to high-traffic areas and green spaces have also been shown to affect asthma outcomes, but were not recorded or available through the American Community Survey. Lastly, we were unable to examine parental factors, including parental health and history of asthma, due to the limited data on parents in the NYC KIDS survey.

### Future Directions

This study revealed that specific characteristics of housing at a population level, including age of buildings and density of apartments within buildings, can impact childhood asthma exacerbations. Neighborhood-level studies and interventions should focus on communities with these characteristics, often a marker for historically less investment in the built environment. Future studies should take a targeted approach and continue to assess which environmental health triggers, such as mold, allergens, and inadequate ventilation drive the greater prevalence of asthma exacerbations among children.

As used in this study, data-driven machine learning techniques can help inform public health practices and policies related to housing interventions to reduce asthma exacerbation among children in NYC. The findings of this study illustrate the significance of neighborhood-level environmental influences that likely contribute to the individual-level residential factors that cause acute asthma exacerbations. The three most influencing drivers in this clustering analysis can aid public health practitioners in targeting interventions at a community level to reduce asthma exacerbation among NYC children. Families who live in neighborhoods with a high proportion of renters, dense apartment buildings, and old building structures are vital areas that could benefit from the prioritization of housing interventions to improve asthma outcomes. Such interventions may include partnering with community-based organizations or Neighborhood Health Action Centers (NHACs) to advocate for community-level interventions and educate providers and families about modifiable, and neighborhood-level risk factors for asthma exacerbations.
